# Improving nutrition and physical activity environments of family child care homes: the rationale, design and study protocol of the ‘Healthy Start/Comienzos Sanos’ cluster randomized trial

**DOI:** 10.1186/s12889-019-6704-6

**Published:** 2019-04-18

**Authors:** Patricia Markham Risica, Alison Tovar, Vanessa Palomo, Laura Dionne, Noereem Mena, Kate Magid, Diane Stanton Ward, Kim M. Gans

**Affiliations:** 1 Center for Health Equity Research, Brown University School of Public Health, Providence, RI 02912 USA; 2 Department of Behavioral and Social Sciences, Brown University School of Public Health, Providence, RI 02912 USA; 3 Department of Nutrition and Food Sciences, University of Rhode Island, Kingston, RI 02881 USA; 4 Department of Nutrition, Gillings School of Global Public Health, University of North Carolina, Chapel Hill, NC 27599-7461 USA; 5 Department of Human Development and Family Studies, University of Connecticut, Storrs, CT 06269 USA; 6 Institute for Collaboration in Health, Interventions and Policy, University of Connecticut, Storrs, CT 06269 USA

## Abstract

**Background:**

Early childhood is a crucial time to foster healthy eating and physical activity (PA) habits, which are critical for optimal child health, growth and development. Child care facilities are important settings to promote healthy eating and PA and prevent childhood obesity; however, almost all prior intervention studies have focused on child care centers and not family child care homes (FCCH), which care for over 1.6 million U.S. children.

**Methods:**

This paper describes Healthy Start/Comienzos Sanos, a cluster-randomized trial evaluating the efficacy of a multicomponent intervention to improve nutrition and PA environments in English and Spanish-speaking FCCH. Eligible child care providers complete baseline surveys and receive a two-day FCCH observation of the home environment and provider practices. Parent-consented 2–5 year-old children are measured (height, weight, waist circumference), wear accelerometers and have their dietary intake observed during child care using validated protocols. FCCH providers are then randomly assigned to receive an 8-month intervention including written materials tailored to the FCCH providers’ need and interest, videos, peer support coaching using brief motivational interviewing, and periodic group meetings focused on either nutrition and PA (Intervention) or reading readiness (Comparison). Intervention materials focus on evidence-based nutrition and physical activity best practices. The initial measures (surveys, two-day observation of the FCCH and provider practices, child diet observation, physical measures, and accelerometer) are assessed again 8 and 12 months after the intervention starts. Primary outcomes are children’s diet quality (Healthy Eating Index), time in moderate and vigorous PA and sedentary PA during child care. Secondary outcomes include FCCH provider practices and foods served, and PA environments and practices. Possible mediators (provider attitudes, self-efficacy, barriers and facilitators) are also being explored. Process evaluation measures to assess reach, fidelity and dose, and their relationship with dietary and PA outcomes are included.

**Discussion:**

Healthy Start/Comienzos Sanos fills an important gap in the field of childhood obesity prevention by rigorously evaluating an innovative multicomponent intervention to improve the nutrition and physical activity environments of FCCH.

**Trial registration:**

(# NCT02452645) ClinicalTrials.gov Trial registered on May 22, 2015.

**Electronic supplementary material:**

The online version of this article (10.1186/s12889-019-6704-6) contains supplementary material, which is available to authorized users.

## Background

Obesity among U.S. preschool aged children (2–5 years) has nearly tripled over the last 30 years [1–6], which is of concern, given that children who are overweight by age 5 are more susceptible to obesity later in life [7], leading to serious health problems [8, 9]. An alarming 22.8% of preschoolers are overweight or obese [5], with low-income and ethnic/racial minority children disproportionately affected [3–5, 10]. Healthy eating and physical activity during early childhood are critical for optimal health, growth and development [11, 12], and poor diets and lack of physical activity increase children’s risk for obesity and related health problems [13–44]. Unfortunately, diet and physical activity patterns of preschoolers, especially low-income and racial/ethnic minorities, often do not meet national guidelines [26, 45]. Effective interventions are needed to improve preschoolers’ food and physical activity environments and behaviors to prevent the development of obesity and related conditions.

Child care settings provide a valuable opportunity to promote healthy eating and physical activity. More than 70% of preschoolers with working mothers are enrolled in out-of-home child care, spend up to 40 h there a week, and consume most of their daily calories while in care [4, 46–49]. Thus, interventions to improve the nutrition and physical activity environments in child care settings are greatly needed [12, 50–54], especially among providers who serve low income, ethnically diverse families.

Among the few reported interventions conducted in child care settings, most have been effective at improving children’s dietary, physical activity and screen-time behaviors [47]; however, all of these studies were conducted in child care centers and none in family child care homes (FCCHs), which care for 25% of US preschoolers [55]. FCCHs have different standards and environments than child care centers, and may not routinely meet best practice guidelines for nutrition or physical activity [31, 56]. Also, children enrolled in FCCHs may be at increased risk for obesity compared to children in center-based care [46]. Thus, obesity prevention interventions in FCCH are critically needed, especially in homes that care for higher risk children. While one randomized obesity prevention intervention trial in FCCH is underway in North Carolina [57, 58], results have not yet been reported, and no studies have included Spanish-speaking family child care providers (FCCP).

This paper describes the interventions, study protocols and measures used in the ‘Healthy Start/Comienzos Sanos’ study (hereafter called Healthy Start). Healthy Start is an innovative multicomponent intervention that engages English and Spanish-speaking FCCPs to improve their food and physical activity-related practices, the nutrition and activity environment of the FCCH, and improves the diet, physical activity and screen-time behaviors of the 2-to-5 year-old children in their care. We hypothesize that FCCP receiving the Healthy Start intervention will have greater changes in nutrition and physical activity practices and environments compared with those in the control group, which received intervention on reading readiness, but no content on nutrition and physical activity.

## Methods

### Study overview, aims and hypotheses

Healthy Start is an ongoing cluster randomized trial with plans to recruit 132 family child care providers (FCCPs) who care for 2–5-year old children. The Specific Aims of the study are to conduct: 1. Formative research to inform the development and adaptation of the FCCH intervention; 2. A cluster-randomized trial to evaluate the efficacy of the nutrition and physical activity intervention among 66 FCCP who receive the Healthy Start intervention and 66 demographically-matched FCCPs who receive a comparison intervention). Primary outcomes include children’s dietary quality, physical activity and sedentary behaviors screen-time at FCCHs. Secondary outcomes include the food, physical activity and screen-time environments of FCCHs and the food and activity-related practices of FCCP. Also to be explored are the relationships between outcome measures and the intervention dose received by the FCCP; the relationship between outcome measures and potential mediating and moderating variables; and the intervention’s effect on the body mass index (BMI) of 2-to-5 year-old children in FCCHs.

Evaluations before and after the intervention include phone and in-person surveys with the FCCP, two-day observations of the FCCH and children’s dietary intake as well as accelerometer and anthropometric measurements of children. After the baseline assessment, FCCH are randomized in matched pairs into either the nutrition and physical activity Intervention group or a Comparison group that receives literacy and reading readiness intervention. The Institutional Review Boards of Brown University, University of Rhode Island and University of Connecticut approved all study procedures and materials. A community advisory board is guiding the study.

### Formative research

To inform the development of the intervention and evaluation protocols, researchers conducted focus groups with FCCPs in Rhode Island. The initial four focus groups included 31 FCCPs; all participants were Hispanic and Spanish speaking; most not born in the US (97%) and nearly half (46%) had a high school degree or less [59]. Providers discussed their perceptions and beliefs regarding what influences physical activity, screen time, and dietary behaviors of the children in their care. They also discussed their own nutrition, physical activity, and screen time practices while caring for children and barriers to meeting best practice guidelines.

FCCPs reported feeling responsible for the health of the children in their care. Although they were aware of the importance of healthy eating and physical activity, implementing these practices was sometimes challenging. These barriers included: costs of providing healthier meals, limited space in FCCHs to encourage more physical activity, finding age-appropriate activities for the wide age range of children in their care, and inappropriate parental beliefs around nutrition and physical activity. Providers agreed that having more resources, activity ideas, and trainings around nutrition, screen-time and physical activity would be helpful to support a healthier FCCH environment. The importance of communicating with parents about healthy eating and physical activity in the FCCH was also considered important [59].

An additional three Spanish-speaking focus groups (*n* = 15) were held later in Rhode Island (RI) with Hispanic FCCPs and one focus group was held in English (*n* = 5). Among these four focus groups two-thirds spoke Spanish only, and 43% had a high school degree or less. Findings were similar to the first four focus groups conducted. The main difference found was that English-speaking providers did not feel that it was their place to change parents’ nutrition and activity-related behaviors and that the child’s home is separate from the child care environment. In contrast, Spanish speaking providers felt like they could play a role in helping to improve parent’s behaviors and the child’s home environment. In addition, there were differences between the Spanish and English-speaking FCCPs in the foods that they reported serving. English-speaking providers reported that they were more reluctant to use spices because the children may not like them and they were more likely to serve “kid foods” like chicken nuggets and hot dogs than Spanish speaking providers who often served Hispanic meals like rice and beans. English speaking FCCPs tended to live in areas with more indoor and outdoor play space and more outdoor play equipment. Thus, playing outside appeared to be a more regular practice in English-speaking than Spanish-speaking FCCH, with the latter discussing the barriers of living on the second or third floor and needing to walk to a park. Also, English-speaking providers were more likely to discuss searching and using internet-based resources such as YouTube videos of dancing and songs for preschoolers. The findings from all 7 focus groups informed the development of the nutrition and physical activity intervention to address attitudes, beliefs and barriers expressed by the FCCPs and also informed the development of the evaluation measures.

Prior to the start of the study, cognitive assessment testing was conducted with all evaluation surveys to assess comprehension, terminology and culturally appropriateness. Six FCCPs from RI were recruited to undergo individual cognitive assessment testing with a bilingual study staff member. The phone baseline survey and in-person baseline survey were each tested individually with two Spanish speaking FCCPs and one English speaking FCCP. Staff members read questions in multiple formats, or asked questions to clarify participants’ understanding of specific word choices in the questions or responses. Participants were also encouraged to ask for clarification of any questions that were unclear. The staff member also asked the participant to explain her reasoning for the response choice she made. The information collected in these sessions was used to inform final revisions to the baseline evaluation measures prior to their use with study participants.

### Recruitment

#### Provider recruitment

The following strategies are being used to recruit FCCPs for the study: 1. Information sessions by research staff members held at community organizations that provide training and support for FCCPs. These organizations also offer recruitment flyers and brochures to FCCPs who come into their offices; 2. Meetings with the coordinators of FCCP systems who then email study information to FCCPs in their systems; 3. Recruitment sessions at conferences organized for FCCPs; 4. Direct mailings followed by staff phone calls to licensed FCCPs whose contact information was available in state databases in Rhode Island (RI), Connecticut (CT) and Massachusetts (MA); 5. Word of mouth referrals from FCCPs already participating in the study. Interested FCCPs are then contacted by research staff to complete a telephone eligibility survey.

#### Provider eligibility criteria

To be eligible for the study, participants must be a FCCP within 60 miles of Providence, RI, have been operating a FCCH for at least 6 months with plans to remain in operation for at least 1 year; read and speak Spanish or English; have a working phone; have at least one 2–5-year-old child in their care for at least 10 h per week, and who eats at least one meal and one snack prepared by the FCCP during their time at the FCCH. In addition, FCCPs cannot plan to close their FCCH for more than 3 consecutive weeks during the year following their enrollment in the study.

### Outcome evaluation protocols and measures

#### Evaluation overview and randomization procedures

Evaluation measures are conducted with each FCCP at baseline, with the same measures administered when the 8-month intervention is completed and again after 4 months (approximately 12 months after the intervention begins). At baseline, eligible FCCPs complete the first part of the baseline survey on the telephone. Upon completion of this survey, time is scheduled for the project field coordinator to conduct an in-person visit at the FCCH. During this visit, the field coordinator completes the enrollment process, which includes review of the study protocols and expectations, completion of the informed consent and implementation of the second part of the baseline survey. The field coordinator also leaves written consent forms for the parents of the age-eligible children in the FCCH. For a child to participate in the evaluation, parents must give consent for their child to have diet observed by project staff, wear an activity monitor and/or undergo anthropometric measurement. When at least one signed parent consent form is obtained giving permission for at least one of the measures, the two-day observation is scheduled with the FCCH.

The two-day observation by research staff members includes measurement of the physical and social environment of the FCCH as well as children’s dietary intake. During these visits, staff members also conduct anthropometric measures of the eligible children whose parents consented. Consented children also wear accelerometers during the 2 days to measure their activity. Evaluation staff are blinded to group assignment to reduce measurement bias. All measures are described in detail below.

Once FCCPs complete the entire baseline survey and in-home observation, they are randomized into either the Intervention or Comparison group. FCCPs are randomized in pairs matched based on their primary language spoken and the number of eligible children within the age range of 2–5 years in their FCCH. The data manager randomly assigns all participants to experimental groups using a Microsoft Excel randomization function after which participating providers are notified of their assignment by a phone call from the project coordinator. Evaluation staff members are not made aware of the intervention group assignment during any aspect of data collection preparation or conduct in the field. Participation in the Healthy Start study, and all data collected through observation or other means are stored in a secure, password protected network or in locked file cabinets.

#### FCCP surveys

The telephone and in-person surveys are conducted in either English or Spanish with the FCCP by trained interviewers and are recorded for quality assurance purposes. Each survey lasts approximately 30 min. All interviewing staff are trained in the use of computer assisted telephone interviewing, which includes a structured set of questions administered identically by each interviewer.

##### Demographics and other FCCP and FCCH characteristics

Demographic information collected includes gender, race, ethnicity, age, marital status, country of origin, years in the U.S., languages spoken at home, household income, household size, subsidized food program participation (Child and Adult Care Food Program (CACFP), the Special Supplement for Women, Infants and Children (WIC) and Supplemental Nutrition Assistance Program (SNAP)), and education. We also query the years as a child care provider, details about other workers at the FCCH, the FCCH hours of operation, number of children cared for in the FCCH overall and by age categories, racial and ethnic proportions of the children, average number of hours children spent daily at the FCCH, languages spoken with the children; FCCP’s use of social media, and intervention video delivery preference (e.g. CD versus online link).

##### Attitudes

The phone survey includes 13 questions to assess provider attitudes about nutrition, physical activity, and screen-time in the child care setting. Providers are asked to express their level of agreement on a 5-point scale (agree a lot, agree a little, neither agree nor disagree, disagree a little, disagree a lot) with a series of statements modified from Lanigan’s Child Care Provider Healthy Eating and Activity Survey [60], our own survey of child care providers [61, 62], and themes that emerged from our focus groups.

##### Self-efficacy

A series of 26 questions on the in-person survey ask the providers how sure they are that they can follow specific practices related to nutrition, physical activity and screen-time in the FCCH to which they respond not at all, a little sure, sure, or very sure. Examples include “how sure are you that you can let the children serve themselves at mealtime?” and “how sure are you that you can always praise and encourage the children for being physically active?” These questions were developed for the current project to align with the child care best practices that informed intervention development.

##### Barriers & facilitating factors

The in-person survey includes 32 questions derived from previous research projects [61, 63], a review of relevant literature, and our focus group findings to assess possible barriers and facilitating factors that might prevent or enable the FCCP to engage in child care best practices related to nutrition, physical activity and screen-time. The items are presented as statements with which the FCCP can express their level of agreement (agree a lot, agree a little, neither agree nor disagree, disagree a little, disagree a lot). Examples of these barrier/facilitating factors include, “You have enough time to sit at the table with the children at meal and snack times,” and, “If you let the children serve themselves, they will waste too much food”.

##### Self-reported practices

Twenty-six questions on the phone survey were derived from the Physical Activity & Diet Behavior with Children in the Home questionnaire [64], developed by Co-Investigator Ward and her team to capture “potentially unobserved” provider nutrition and physical activity practices during an intervention study with FCCHs in North Carolina [57, 58]. Twenty-one questions include: the self-reported frequency of practices (never, rarely, sometimes, often, very often, or always) related to role-modeling, self-regulation, using food as a reward, screen time during meals, and praise and encouragement for healthy eating. Additional items assess agreement (agree strongly to disagree strongly) with statements about seeking professional training and provider communication with parents and children regarding healthy eating and physical activity.

The phone survey also includes self-reported practice questions modified from the validated Nutrition and Physical Activity Self-Assessment for Child Care (NAP SACC) tool [65], which is based on best practice recommendations. Eight questions assess how often (per week, day, or month) a provider offers fried meats, fried potatoes, other fried foods, high-fat meats, sweets, salty snack foods and 100% juice. Final responses are calculated into frequency per week. For juice, we also collect the typical amount offered each time. Two other questions ask how often providers lead planned nutrition education and physical activity activities for children (rarely or never, 1 time per month, 2–3 times per month, 1 time per week or more). Two questions ask about the average daily minutes the children spend watching TV and screen time on other devices. Also, three question sets ask if the provider shares specific topical information with families about nutrition (6 topics), physical activity (5 topics), and screen-time (3 topics) with response for each offered as yes or no. These questions are used for evaluation purposes as well as to create tailored intervention reports for FCCPs in the Intervention group.

##### Questions to assess comparison group outcomes

The in-person survey includes 20 reading readiness questions modified from Get Ready to Read’s Family Child Care Literacy Environment Checklist. These questions ask the FCCP the frequency (never, rarely, sometimes, often, always) by which they incorporate various literacy activities with children in their FCCH. The literacy practices include playful use of words and writing, exposure to new words, and reading with the children.

##### Two-day FCCH observation and child measurements

Upon completion of both the phone and in person survey, FCCPs undergo 2 days of observation and child measurement in their FCCH at each evaluation time point. The two observation days are scheduled at the convenience of the FCCP, as well as anticipated availability of the consented children. Staff members arrange with the FCCP to arrive before the children eat their first meal or snack at the FCCH. Observers position themselves in order to observe up to 3 children in a convenient location to avoid interfering with the daily routine. If more than 3 children are consented to participate, two or more research staff members conduct the observation. Observers leave the FCCH during the children’s naptime and return to continue with observation until the children leave the FCCH to go home. The observation includes the Environment and Policy Assessment and Observation (EPAO) and the Dietary Observation in Child Care (DOCC). The EPAO was developed and validated by Co-Investigator Ward and her team to evaluate observed practices, environments, and policies within child care centers and FCCHs that influence children’s nutrition, physical activity, and sedentary behavior [66]. DOCC is a reliable, valid visual observation technique for measuring children’s dietary intake also developed by Co-Investigator Ward and her team [67, 68].

During the two-day observation, research staff also measure consented children’s height, weight, and waist circumference and place the accelerometers on the consented children. All field staff conducting observations and measurements undergo rigorous training and certification.

##### Environment and policy assessment and observation (EPAO)

Food physical environment measures include types and frequency of food and beverages available and served and other physical supports for eating Food social environment measures include FCCP feeding practices, guidelines, encouragement, role modeling, nutrition education offered to children and parents, and supportive or unsupportive behaviors related to healthy eating. Physical activity and screen-time physical environment measures include active and sedentary play opportunities inside and outside; TV or videos in the room and used by children or FCCP; types of active play equipment and toys inside and outside; availability of play space inside and outside, outdoor play time; and presence of posters, pictures, books about active play. Social physical activity and screen-time environment measures include FCCP behaviors that are supportive or unsupportive of physical activity and screen-time, and education to children and parents about physical activity or screen-time.

The EPAO was adapted to reflect cultural differences for the participant population based on formative research. For example, we added food items such as plantains, yautia, and yucca as starches in the “Potatoes” section and “other sugary drinks” to the “Beverages” section to capture sugary homemade beverages. We also added an “other fried foods” category to capture fried foods such as empanadas that would not fit in the existing categories. We updated the EPAO to include all possible food items at every meal/snack because focus group participants talked about sometimes feeding children more of a “dinner” meal as an afternoon snack or a “breakfast” meal as a morning snack, if they were concerned about the children not eating those meals at home.

The observer records detailed notes about the environment and the FCCP’s behaviors as they relate to nutrition, physical activity and screen-time during the home visit. Forms are reviewed for accuracy and completeness by field staff, and then submitted to data staff for an additional review. All forms are scanned for electronic data capture with Teleform software, and verified again for accuracy of the electronic data.

##### Dietary observation in child care (DOCC)

Children’s food intake is measured during the two-day observation using the DOCC system [67]. Observation is the gold standard for measuring children’s diets and is far more accurate than recall by provider or child [69–71].

Trained and certified research staff observers visually estimate and record the amount and type of foods and beverages that FCCPs serve to each consented child at meals or snacks. Foods not easily discernible are clarified with the FCCP (e.g., type of milk, added fat, food preparation information and brand names). For more complex recipes or mixed dishes, observers ask FCCPs for the recipe and preparation information. Observers carefully watch the amount of food wasted (e.g., dropped or traded, etc.). At the end of the meal or snack observed, research staff estimate the amount of food remaining. The amount of food consumed is estimated as the amount served minus the amount wasted or remaining. Food observation data is then entered into the Nutrition Data System for Research (NDSR) (Nutrition Coordinating Center, (NCC), Minneapolis, MN) to calculate dietary outcomes. We check the validity and reliability of all observers (field staff plus two Registered Dietitians (PMR and NM) based on the techniques described by the UNC team) [67]. The certification process includes a lab component, during which the field staff must accurately estimate at least 80% of 20 measured portions of typical child foods. After passing the lab certification process, field staff must also achieve 80% inter-rater reliability with a “gold standard” observer in the field at a FCCH. DOCC observers must pass the certification process annually, as well as participate in structured monthly practices, quarterly validity checks, and semi-annual inter-rater reliability checks.

##### Accelerometer measurement of children’s physical activity

The accelerometer is an objective measure of children’s physical activity in the FCCH measuring the amount of time each child spends in sedentary, light, moderate, and vigorous activity across the 2-day observation period, excluding nap time. At the start of each day, research staff members place an accelerometer on a belt around the waist of the consented children. For most children accelerometers are worn all day, removed by research staff members before the child leaves to go home. The accelerometer is worn during nap time unless the child complains of discomfort, in which case it is removed and put back on when the child wakes up. Dr. Ward’s protocols and evidence-based guidelines for accelerometer use in preschool children are followed [68, 72–74]. After children have worn the accelerometer for both observation days, the accelerometer data are uploaded to an office computer for processing.

##### Anthropometric measurements of children

Research staff use standard techniques for measuring height, weight, and waist circumference of consented children [75]. The research staff member conducting measurements sets up equipment in a space visible to, but located away from the main activities. Children who assent come to the area to be measured one at a time. Height is measured using a SECA portable stadiometer to the nearest 8th of an inch. Weight is measured using a Tanita digital scale to one decimal place. Waist circumference is measured to the nearest 8th of an inch by holding a standard tape measure around the child’s waist parallel to the floor at the top of their right ilium according to the CDC NHANES protocol [76]. The series of three measurements is repeated a total of 3 times and averaged for each child.

#### Training of field staff

Field staff members undergo an intensive multi-day training in the lab to cover the instruments, observation procedures, and record keeping for the EPAO by Dr. Ward’s UNC team. Trainers use a comprehensive training manual and discuss example scenarios that might be encountered. The lab training includes videos of different feeding practice scenarios to train the data collectors on how to assess provider feeding practices. After the in-house training, the staff members shadow an experienced observer for a day in an actual FCCH. Both the experienced observer and staff trainee complete an independent full-day EPAO observation with record keeping. After the observation, the staff trainee’s records are discussed with the trainer. EPAO certification is conducted with field observers prior to independent data collection with a requisite 85% agreement between each observer and the gold standard observer. Field staff members communicate weekly with the data manager via in-person discussion, meetings, notes and emails to discuss any unusual scenarios and/or to revisit best practices for observations and form completion. Field staff members also undergo training and certification for measuring anthropometrics and placing the accelerometer on children.

Healthy Start team registered dietitians (PMR and NM) were trained and certified by Dr. Ward’s team to provide training for the field staff on DOCC observation of children’s food intake. The DOCC training includes in-house lessons on visual serving size estimation, proper food measurement techniques, general observation techniques and proper form completion. The training process typically lasts several weeks before field staff members begin the certification process according to the established protocol [67]. As described above, validity is checked between observations compared with measured food portions. Reliability is assessed between the field observers with the, “Gold Standard,” observer within our group (NM).

Financial incentives are provided to FCCP throughout the study to compensate for their time and inconvenience for evaluation measures. FCCP are provided with a $25 gift card at every in-person survey meeting and $50 on the second day of each two-day observation. Also, at the conclusion of the study, FCCP are given $365 as an overall, “Thank you,” for participation and for any possible disruption to their business, for a total of $590 possible incentives.

### Interventions

#### Theoretical framework

**Fig. 1:**
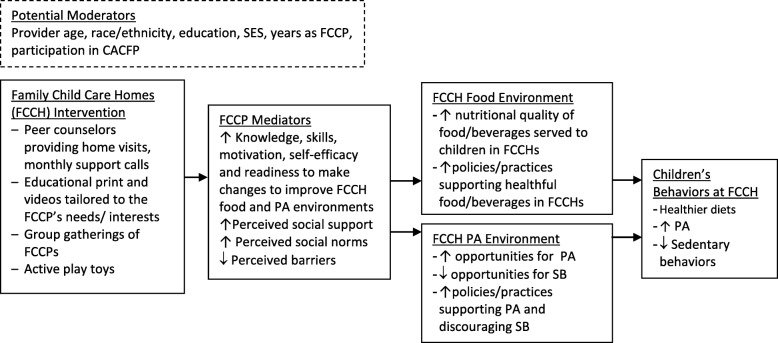
Intervention logic model

The Healthy Start intervention is informed by the social ecological framework [77–81], which recognizes that behavior is affected by multiple levels of influence and that interventions are most effective when they target changes in intrapersonal, interpersonal (social), and environmental domains [77, 81–84]. The intervention operates at all of these domains and is also informed by Social Cognitive Theory (SCT), which defines behavior as a dynamic and reciprocal interaction of personal factors, behavior and the environment [85–89]. The intervention targets key components of the SCT to change FCCPs’ behavioral capability, self-efficacy, outcome expectations, and perceived social support, norms and barriers that will in turn lead them to improve their food and activity-related practices and their FCCH’s environment to better support healthy eating and physical activity. Moreover, motivational interviewing used by the support coaches, are expected to increase FCCP’s motivation and readiness to change as posited by Self Determination Theory [90, 91]. Positive behavior changes of the FCCP are expected to result in changes to the environment of the FCCH that will in turn lead to improvements in children’s diet, physical activity and screen-time behaviors. (See Logic Model in Fig. 1).

#### Intervention overview

For both the Intervention and Comparison group, the eight-month intervention includes four components: support from a lay coach, tailored written materials, videos, and an in-person group meeting; however, the content is specific to experimental group. FCCPs are assigned a lay support coach who has been trained in either the nutrition/physical activity (Intervention group) or literacy/reading readiness (Comparison group) content. The intervention begins with an in-person visit at the FCCH led by the support coach. The support coach reviews with the FCCP an individually tailored written feedback report generated by our computer-based expert system based on the results of the baseline surveys and observations. The coach then conducts brief motivational interviewing (MI), called motivational enhancement (ME) [92] with the FCCP. At the end of the ME session, the FCCP selects topics to work on (1 for each month, 8 in total).

Approximately 2 weeks after this in-person session, the FCCP receives a newsletter in the mail and a 3–6-min DVD (or emailed video link) with information related to the first topic selected. The newsletters are available in English and Spanish, written at a sixth-grade reading level and designed to be interactive, with checkboxes and options for the FCCP to mark ideas they want to try or to list new ideas. Approximately 2–3 weeks later, the support coach calls the FCCP and again uses ME to discuss progress, and to help the FCCP select the next topic to work on. For the next 7 months, FCCPs are sent a monthly newsletter and video based on the topic they chose to work on that month. Support coach ME-based phone calls follow each mailing. In addition, group meetings are held approximately every 6 weeks separately for the Intervention and Comparison groups in a central public location. All participating FCCPs are invited to attend these meetings, led by the support coaches, to discuss challenges and successes, learn a new activity, and share a meal. Details are provided below about each intervention component.

#### Support coach recruitment and training

A total of six support coaches work on the Healthy Start project. All coaches are bilingual (English and Spanish) community health workers with previous experience in child care, education, nutrition, and/or child health. Coaches were recruited through partnerships with the Community Health Worker Association of Rhode Island and Ready to Learn Providence and selected based on their previous experience as community health workers and their potential to learn and execute the intervention.

Support coach ME training sessions are conducted over 16 h by a Brown faculty member with a PhD in Psychology and extensive experience in health behavior change and MI. Support coaches are trained on the general principles of ME, and then specific skill areas within ME. The applied skills include open ended questions, affirmations, reflections and summaries, active listening, problem-solving, assessing readiness to make a change, and handling resistance.

After being trained in ME, support coaches are then chosen to work as coaches for either Intervention or Comparison group depending on their background and experience. Three coaches receive training on the best practices related to nutrition, physical activity, and screen-time for the Intervention group and three on the best practices related to early literacy skills and reading readiness for the Comparison group. Each group of coaches receives separate training on the specific topical protocols for implementing the in-person intervention visits and monthly phone calls with the FCCPs in their assigned experimental group. The protocol includes selecting a topic for the provider to work on, problem solving/goal setting, assessing barriers, assessing readiness to make a change, and recording change talk (DARN: Desire, Ability, Reason, Need) throughout the session with the provider. In addition to classroom training, support coaches also conduct mock sessions both in the classroom and at home. Sessions are recorded and reviewed by the instructor. Mock practice continues until minimum protocol standards are achieved. Support coaches receive ongoing supervision, and monthly feedback and training related to ME skills from the clinical psychologist to ensure that they follow protocols throughout the study.

#### Nutrition and physical activity intervention components

##### Initial tailored report

Based on data gathered from the FCCP surveys and the 2-day observational assessment in the FCCH, we created a computerized expert system with algorithms that determine whether FCCPs meet a variety of best practices derived from the NAP SACC assessment [65, 93, 94]. The computer algorithms compare the FCCP’s current practices (based on baseline data) to the best practices for 22 topic areas including specific foods and beverages offered to the children, FCCP feeding practices, mealtime environment, role-modeling, encouraging healthy behaviors, physical activity opportunities, screen time limits, education provided to parents and children, and policies and parent communication related to nutrition and physical activity. The algorithm compares observed behaviors from the EPAO to the best practice whenever possible. For example, the best practice for milk is that children ages 2 and older should only be served skim or 1% milk. The algorithm determines that the FCCP meets the best practice if the staff observer indicates that provider does NOT serve 2% or whole milk at any meal or snack time. However, because some best practices refer to a timeframe that goes beyond the project’s two-day observation period, both observation and self-report data from the survey questions are used in the algorithm for certain best practices. For example, the best practice for juice intake is to limit 100% fruit juice to no more than two 4–6 oz servings per week. The algorithm determines that the FCCP meets the best practice if the staff observer indicates that the total amount of 100% fruit juice served to a child across the 2 days of observation does not exceed 12 oz; AND the amount of 100% fruit juice that the provider reports serving the children does not exceed 12 oz per week (See Table 1).

**Table 1:** Best practices from NAP SACC and algorithm for meeting best practices based on survey responses and/or EPAO data

Domain	Best practice	Requirement to meet best practice	N = Newsletter V = Video	
Water	Make drinking water available for children at all times.	Staff observer indicates that children have self-service access to water in the FCCH (including from filled cups that are always accessible)	Water	Drink Water!
Water	Prompt children to drink water during each indoor and outdoor play time.	Staff observer indicates that provider reminds children to drink water at least once during every outdoor play time and every active indoor play time.		
Juice	Limit 100% fruit juice to no more than two, 4-6 oz. servings per week.	Staff observer indicates that the total amount of 100% fruit juice served to a single child across the 2 days of observation does not exceed 12 oz; AND the amount of 100% fruit juice that the provider reports serving the children does not exceed 12 oz per week.	Juice	Juice
Juice	Only serve 100% fruit juice that has no sugar added.	Staff observer indicates that provider does not serve juice that is less than 100% fruit juice at any meal or snack time.		
Milk	Children ages 2 and older should only be served skim or 1% milk.	Staff observer does NOT indicate that provider serves 2% or whole milk at any meal or snack time.	Milk	Milk
Milk	Never serve flavored milk (milk with chocolate or strawberry syrup, or with added sugar).	Staff observer does NOT indicate that provider serves flavored milk at any meal or snack time.		
Sugary Drinks	Never serve sugary drinks.	Staff observer does NOT indicate that provider serves sugary drinks (e.g. fruit flavored drink, lemonade, sports drink, soda, sweetened tea, or homemade drink with added sugar) at any meal or snack time.	Sugary Drinks	Sugary Drinks
Vegetables	Offer children vegetables two or more times a day.	Staff observer indicates that provider offers vegetables at more than one meal or snack time on each observation day.	Vegetables	Vegetables! How you can serve more
Vegetables	Don’t prepare vegetables with added fat. Small amounts of vegetable oil is the healthiest option.	Staff observer does NOT indicate that vegetables are fried or prepared with lard, butter, margarine, or cheese sauce at any meal or snack time.		
Fruit	Offer children fruit two or more times a day.	Staff observer indicates that provider offers fruit at more than one meal or snack time on each observation day.	Fruit	Fruit!
Fruit	Never serve fruit in syrup or with added sugar.	Staff observer does NOT indicate that fruit served at any meal or snack time was canned in syrup or sweetened with added sugar.		
Whole Grains	Offer children high fiber, whole grain foods two or more times a day.	Staff observer indicates that FCCP offered a whole grain food (including whole grain breads, pastas, cereals, crackers, and granola bars) two or more times daily on both observation days (at any combination of morning meal, morning snack, lunch, and afternoon snack)	Whole Grains	Whole Grains
Snack Foods	Limit offering children sugary, salty, or fatty foods to less than 1 time per week or never.	Staff observer indicates that the provider does NOT serve crackers, pretzels, chips, dessert items, sugary cereal, granola bars, pastries, or Poptarts at any meal or snack time; AND the provider reports serving such items less than once per week.	Healthy Snacking	What are Healthy Snacks for Children More Healthy Snack Ideas
High-fat meats	Limit serving high-fat meats to less than 1 time per week or never.	Staff observer indicates that the provider does NOT serve bacon, ham, hot dogs, bologna, salami, regular sausage, or other high-fat meat at any meal or snack time; AND the provider reports serving such items less than once per week.	High Fat Meat	Healthy Meat Options
Fried and Pre-Fried Foods	Limit offering children fried or pre-fried foods to less than 1 time per week or never.	Staff observer indicates that the provider does NOT serve fried meat, fried potatoes, or other fried foods at any meal or snack time; AND the provider reports serving such items less than once per week.	Fried Foods	How to Avoid Fried Foods
Mealtime Environment	Always sit at the table and eat with the children.	Staff observer indicates that FCCP sat with the children “a lot” at every observed meal on both days (morning meal, morning snack, lunch, and afternoon snack)	Mealtime Environment	Mealtime Environment
Mealtime Environment	Teach children how to serve themselves or, in the case of older children, allow them to serve themselves.	Staff observer selected EPAO option that “children served themselves most or all foods, and decided what size portions to take” at every observed meal and snack time. This best practice could still be met if a staff observer indicated for a given meal that “food was brought from home” or that “children served themselves most or all foods, and decided what size portions to take. Provider served fruits and/or vegetables”.		
Self-Regulation	Always ask children if they are full before removing an unfinished meal or snack plate.	Staff observer indicates that provider never removes an unfinished plate without asking a child if they are full at any observed meal and snack time.	Self-Regulation	Hungry or Full: Paying attention to Our Bodies
Self-Regulation	Always ask children if they are hungry before serving more food.	Staff observer indicates that provider serves seconds only after a child requests them and after asking if child is still hungry “a lot” at every observed meal and snack time where seconds are served.		
Self-Regulation	Never pressure children to eat more food than they want.	Staff observer indicates that provider never required a child who ate less than half of a meal or snack to sit at the table until they cleaned their plate at any observed meal or snack time.		
Self-Regulation	Do not use food or sweets as a reward or reward children for finishing their plate.	Staff observer indicates that provider never uses food or sweets as a reward or rewards children for finishing their plate at any meal or snack time.		
Role Modeling	Enthusiastically role model eating and drinking healthy foods	Staff observer indicates that provider enthusiastically role models eating and drinking healthy foods a little, sometimes, or a lot at 75% or more of observed meal and snack times.	Role Modeling	Be a Role Model
Role Modeling	Always participate in indoor physical activity with the children.	Staff observer indicates that provider plays actively with children an average of “a lot” during morning and afternoon observed indoor playtime across both observation days.		
Role Modeling	Always participate in outdoor physical activity with the children.	Staff observer indicates that provider a) joins children in a game, b) plays with children, and c) joins a chasing/running game with children an average of “a lot” during morning and afternoon observed outside time across both observation days.		
Role Modeling	Do not model sedentary behavior.	Staff observer indicates that provider does not watch any TV or use other screen time during observation period.		
Encouragement	Always prompt and praise children for trying new or less preferred foods.	Staff observer indicates that provider prompts and praises children for trying new, less preferred, or healthy foods a little, sometimes, or a lot at 50% or more of observed meal and snack times.	Encouragement	Encouragement
Encouragement	Always prompt and praise children for being physically active.	Staff observer indicates that provider a) praises a child for being physically active at least “a little” and b) prompts children to increase their physical activity at least “a little” each observed morning and afternoon.		
Nutrition Education	Lead a planned nutrition education lesson one or more times per week.	Provider reports leading a planned nutrition education lesson at least once per week.	Nutrition Education Activities	Nutrition Education: Teach Explore Eat!
Nutrition Education	Talk with children informally about nutrition and healthy eating as often as possible.	Staff observer indicates that provider talks with children informally about nutrition a little, sometimes, or a lot at every observed meal and snack time.		
Parent Communication	Provide families with information on child nutrition to help them continue healthy practices at home.	Provider reports sharing information with families about child nutrition topics, including 1) types of foods and drinks children should eat, 2) recommended serving sizes, 3) the importance of serving a variety of foods, and 4) a healthy mealtime environment.	Parent Communication	Parent Communication
Parent Communication	Provide families with information on children’s physical activity to help them continue healthy practices at home.	Provider reports sharing information with families about child physical activity topics, including 1) the amount of time children should spend being physically active, 2) encouraging children to be physically active, 3) limiting long periods of seated time, 4) the amount of time children should spend playing outdoors, and 5) using the outdoors to encourage children’s active play.		
Parent Communication	Provide families with information on children’s screen time to help them continue healthy practices at home.	Provider reports sharing information with families about child screen time topics, including 1) the amount of screen time children should have, 2) why it’s important to limit screen time, and 3) activities children can do instead of screen time.		
Physical Activity	Provide children with 90 min or more of indoor or outdoor play PA each day.	Staff observer records 90 min or more each day during which time children engage in activities at least as intense as walking or marching.	Physical Activity	Physical Activity for All Seasons
Physical Activity	Provide children with 60 min or more of outdoor play each day.	Staff observer records 60 min or more each day during which time children spend outdoors.		
Adult Led Physical Activity	Provide children with 45 min or more of adult-led physical activity each day.	Staff observer records 45 min or more each day during which time an adult leads children in physical activity.	Adult Led Physical Activity	Adult-Led Play
Sedentary Time	Limit the time children are asked to remain seated on any occasion to less than 15 min at a time.	Staff observer does not indicate any occasion when children are required to sit for more than 15 min at a time during the 2 days of observation.	Sedentary Time	No Video
Screen Time	Limit children’s screen time to less than 30 min per week.	Staff observer records less than 30 min of children’s screen time across both observation days; AND the amount of children’s screen time that the provider reports allowing is less than 30 min per week.	Screen Time	Screen Time
TV at Meal Time	TV is never on during mealtime or snack time.	Staff observer indicates that children do not use the TV or other screen device during any observed meal or snack time.		
Physical Activity Education	Lead planned physical activity class one or more times per week.	Provider reports leading a planned physical activity lesson at least once per week.	Physical Activity Education	Physical Activity Education: Teach Explore Move!
Physical Activity Education	Talk with children informally about the importance of physical activity.	Staff observer indicates that provider talks with children about the importance of physical activity at least “a little” at any time during the observation days.		
Policies	Establish a set of nutrition and physical activity policies in place for your child care. Share these policies with parents so they understand the practices in your child care.	Staff observer indicates that provider has written policies about nutrition, physical activity, and screen time in place for their FCCH.	Policies	Policies for Your Childcare

The computerized expert system uses the algorithms for the 22 topics to generate an individually tailored feedback form for each FCCP. If the FCCP currently meets a best practice, a green check mark is shown on the feedback form as a positive sign of success in that area. If the FCCP does not currently meet the best practice for an item, the space is left blank on the form, indicating an opportunity for improvement. For example, the “Whole Grains” topic area consists of a single best practice – to offer children whole grain foods 2 or more times each day. If the staff observer indicates that the FCCP offered a whole grain food two or more times daily on both observation days (at any combination of meal and/or snack) they receive a green check mark on the tailored feedback report. This feedback report is provided to the FCCP during the initial home visit with the support coach. See example of a tailored feedback report in Additional file 1: Figure S2.

#### Initial home visit with support coach

During the first in home session, the support coach reviews the tailored feedback form with the FCCP. Then, using the ME protocol, the support coach identifies the FCCP’s readiness to make a change, helps the FCCP set a goal related to one topic, identifies potential barriers to completing the goal, and discusses plans to overcome those barriers. The FCCP also documents her level of motivation and confidence to make this change (on a scale of 0–10). Throughout each of these steps, the support coach creates a collaborative environment that is supportive of the FCCP’s own autonomy and self-efficacy. The support coach listens to the FCCP’s responses and reflects back the change language that she hears the FCCP say.

The support coach documents the results of the session on a data form including the topic the FCCP chose to work on, the specific goal for the topic, any stated barriers to achieving the goal, the FCCP’s level of motivation and confidence for achieving the goal, and any, “change talk,” the support coach heard during the conversation about the FCCP’s readiness and reasons to make the change. The coach also records the date and time of the phone call for the following month. This process is repeated each month for 7 additional months during the monthly phone calls with the FCCP and support coach See Support Coaching by Phone.

At the end of the visit, the Intervention group FCCPs receive a set of active play toys to use with the children, including “pool” noodles, hula hoops and foam hoop holders, a tunnel, bean bags, soft balls, feather-light balls, and plastic colored spots. The toys are also accompanied by a set of videos and activity cards that demonstrate activities utilizing the toys.

#### Tailored newsletters

For each of the 8 months of the intervention period, participants receive a tailored newsletter covering a different theme based on the topic areas they selected during the initial home visit and/or the phone coaching sessions and the content of those conversations. The first newsletter packet, mailed 1–2 weeks after the in-person visit, begins with a cover letter that is individually tailored with the name of the FCCP, the name of their support coach, the FCCP’s topic choice (chosen during the support coach home visit) and the FCCP’s current practices related to this topic (based on data taken from the surveys and/or observation). This tailored letter also reminds the FCCP of the time for their upcoming scheduled support coach call, and includes a brief description of the FCCP’s expressed barrier, goal for the month, and comments gathered from the last conversation with their support coach. The rest of the newsletter packet includes 4–5 pages of content about the FCCP’s chosen topic (See main topics in Table 1). If the FCCP mentioned a barrier related to this topic during the conversation with the coach, they also receive a barrier page providing suggestions on how they might overcome the barrier. Barriers covered in newsletters include those most commonly mentioned in our formative research including time, cost, taste, anticipated mess, resources needed and others. (See an example newsletter in Additional file 2: Figure S3).

#### Videos

Along with the mailed newsletter, FCCPs also receive a short, 3–5-min video related to their chosen topic with audiovisual content in the language of their choice (English or Spanish). The videos show actual FCCPs demonstrating best practices with the children in their care with simple, straight-forward tips and explanations for making changes. Segments include: testimonials from other FCCPs, cooking demonstrations of healthy recipes, scenarios displaying problem-solving, and demonstrations of activities to do with children. FCCPs choose to receive the videos in one of three ways: 1) A mailed DVD; 2) An emailed link to view the video online; 3) A text message containing a link to view on their smartphone.

#### Support coaching by phone

After receiving their first tailored newsletter, FCCPs receive monthly phone calls (approx. 20–25 min) with their support coach for the next 7 months. At the start of each phone call, support coaches ask if the FCCP received their newsletter and video, and if they read or watched the material. Then, the support coach again uses the ME approach to discuss thoughts and concerns and to check in on the FCCP’s progress in completing their goal and assist in addressing barriers. The FCCP also selects a new topic for the next month, establishes a goal and discusses possible barriers. The support coaches again listen for “change talk” from the FCCP including reasons they want to make a change. The support coaches document the conversation on a data form, including notes about the completion of goals for the previous month, responses about the usefulness of the previous newsletter and video (from a scale of very useful to not at all useful), the selected new topic, and associated goal, barriers, change talk and motivation and confidence (on a scale of 0–10). This data form is then incorporated into the computerized expert system to generate the next tailored mailing (cover letter, newsletter and video) to be sent to the FCCP. All phone calls between the FCCP and the support coach are recorded for quality assurance purposes.

#### Group meetings

Support group meetings are held approximately every 6 weeks at a local community location such as a library or church. FCCPs in the Intervention group are invited to attend the meetings led by the Intervention group coaches. The meetings, which last approximately 90 min, include a light meal and demonstration of an activity related to nutrition and/or physical activity education that FCCPs can do with the children in their care. The meetings also serve as a time for social support where FCCPs discuss challenges and solutions they face while working on different monthly topics. Support coaches are present and lead the activities, but mainly serve as facilitators.

#### Intervention components for the comparison group

FCCPs randomized into the Comparison group receive the same intervention components, dose and intensity as those in the Intervention group, except the content is related to reading readiness and early literacy skills. As with the Intervention group, the Comparison group components include the tailored feedback form, the in-person home visit and seven monthly calls from the support coach using ME, 8 tailored newsletters and videos mailed monthly, and the group meetings (approximately every 6 weeks), with content related to reading readiness and early literacy skills rather than nutrition and physical activity. The FCCP selects which of eight intervention topics related to reading readiness they want to work on each month and when they hope to begin. As with the intervention group, they get a packet starting with a tailored cover sheet, however, they do not receive individually tailored content within the newsletter packet nor barrier pages. The intervention content was adapted from the Reading Rockets and Coloring Colorado curriculum materials [95–97]. Materials were reviewed and revised as needed by experts in early childhood education at the University of Connecticut. Topics for the newsletters and videos include why reading is important, getting ready to read, reading aloud, wordless picture books, building vocabulary, early writing skills, interactive play, and creating a home library. Instead of a set of activity toys, FCCPs in the Comparison group receive a set of 10 books including 3 wordless pictures books. FCCPs can choose to receive books in English and/or Spanish.

### Evaluation and analysis

The Healthy Start evaluation includes process evaluation measures to assess intervention delivery and satisfaction as well as effect evaluation of outcome and behaviors to assess changes in primary, secondary and exploratory outcomes.

#### Process evaluation and analysis

Process evaluation measures assess participant satisfaction and fidelity of intervention delivery, including staff adherence to intervention protocols and dose of the intervention received by participants.

##### Participant satisfaction

Participant satisfaction is assessed on the 8- and 12-month follow-up surveys. Questions assess the perceived helpfulness of all intervention components including the support coach, videos, newsletters, group meetings, toys and toy video and activity cards (or books for the Comparison group). Questions also include how much the providers feel that the newsletters were created just for them and were easy to read and understand. FCCPs are also asked how well the support coach helped them to make changes in their FCCH and respected efforts to improve their FCCH. Providers in both groups are asked what they liked most, what they liked least, and how the Healthy Start program can be improved. They are also asked to list changes they have made in their FCCH and changes to their own nutrition, physical activity, or screen time habits because of the Healthy Start program. In addition to the survey questions FCCPs who attended the group meetings fill out a brief evaluation form at the end of the session, which queries helpfulness and usefulness of the sessions, what they liked about the group meeting, possible improvements to be made, and convenience of the location and time.

##### Fidelity

Adherence to the intervention protocol is measured in several ways. Project staff track mailings of the newsletters and videos as well as implementation of group meetings. Attempts to connect with FCCP as scheduled are recorded by support coaches; these logs are used to assess coaches’ effort to connect with FCCPs. Support coaches also complete forms to document what was discussed on each call. Support coaches record all phone calls; at least 10% of the recordings with FCCPs are reviewed to assess ME skills and fidelity with subject content.

Dose is measured as the number of newsletters and videos read and watched, group meetings attended, and toys/books used as reported by FCCP on the 8- and 12-month surveys. Additionally, the software used to send video links to FCCP via email or text tracks if the link was clicked; the website that hosts the videos online also tracks how many times a video is watched by the FCCP. Support coach call tracking records enumerate attempts to reach FCCP and call completions, as well as the length of each completed call. Support coaches or staff members facilitating group meetings record FCCP attendance. Additionally, providers are asked about any additional training they received outside the study related to nutrition or physical activity to evaluate potential contamination between comparison and Intervention groups.

Process evaluation data will be quantified as counts and proportions for each intervention component. Relationships between intervention dose and outcome measure changes will be studied using linear regression models.

#### Effect evaluation and analysis

##### Sample size considerations

The proposed final sample size of 60 FCCHs per experimental group with 3 children per site was chosen to detect differences in primary outcomes consistent with changes found in our Healthy Homes, Healthy Families pilot study, as well as effect sizes in Dr. Ward’s and other published research in child care centers [47, 49, 93, 98]. We anticipate that our proposed final sample of 60 FCCHs per condition with power of 0.8 or greater and alpha of .05 will allow for detection of effect sizes of at least 0.25 serving of fruits and vegetables per day and 3 oz of 100% fruit juice or sugary beverage, as well as increase of 2.1% time in moderate/vigorous physical activity or 2.2% screen-time. To account for expected attrition of 10%, we will recruit 66 homes in each experimental group (total *n* = 132).

#### Exploration of group assignment

Demographic and other baseline characteristics will be compared between groups to confirm that the random assignment resulted in statistically similar groups. Any variables found to be different between groups at baseline will be included in final models. Child-level outcomes will be adjusted for clustering at the FCCH level using generalized estimating equation (GEE) models with exchangeable correlation matrices.

##### Primary outcomes

Our primary outcomes are changes in children’s dietary quality and changes in children’s physical activity. Dietary quality is measured using the 2015 Healthy Eating Index (HEI) score, which is calculated based on data collected during the two-day observation using the DOCC. DOCC data collected in the field are reviewed for completeness and entered into the NDSR. The NDSR data are then inputted into Statistical Analysis Software (SAS) (SAS Institute, Cary, NC) to calculate HEI component scores and total HEI-scores. The HEI components scores include total fruit, whole fruit, total vegetable, dark green and orange vegetables and legumes, total grain, whole grain, milk, meat and beans (adequacy) and saturated fat, sodium, added sugars, and solid fats (moderation). These components are derived using established scoring methods written as publicly available USDA SAS codes [68]. SAS codes add food group equivalents and divide over 1000 cal, creating a normalized variable per adequacy or moderation component. Adequacy components are positively scored, where a higher intake results in an increased score, while moderation components are reversed scored, where a lower intake results in an increased score. Total HEI scores are generated by combining adequacy and moderation scores for a maximum score of 100.

Children’s physical activity is measured using accelerometer data. After being removed from the children in the FCCH, accelerometers are brought back from the field and entered into the computer to be scored in batches with the Actilife software (Actigraph [99]), using appropriate cut points and energy expenditure formulas for 2–5 year old children. [100, 101]. Time filters are applied based on the times that field staff record affixing and removing the accelerometers each day, as well as the times the children begin and end their naps with or without wearing the device. Scored data files describe the amount of time each child spends in sedentary, light, moderate, and vigorous activity across the 2-day observation period, not including naptime.

##### Secondary outcomes

Secondary outcomes include changes in FCCH practices from the pre-post surveys and the EPAO and changes in FCCH nutrition and activity environments from the EPAO. This analysis will assess the overall EPAO nutrition score and subscales created by the UNC originators of the tool [102].

##### Exploratory outcomes

Using measured height and weight, changes in children’s BMI and BMI z score and BMI percentile will be calculated for children at all time-points. We will look at both mean values and at-risk categories based on CDC guidelines [103]. Changes in waist circumference will also be assessed and compared to estimates for normal and at-risk categories.

#### Analysis plan

Initially, descriptive statistics will be used to summarize the variable and detect outliers, data entry mistakes and missing values, including exploratory graphical techniques. The normality of the distribution of primary and secondary outcomes, both in their original and transformed state (if necessary), will be examined with a normal probability plot. Demographic and other baseline characteristics will be compared between groups to confirm that the random assignment resulted in statistically similar groups. Any variables found to be different at baseline will be included in adjusted models. Child-level outcomes will adjust for clustering at the FCCH level using GEE models with exchangeable correlation matrices. We will explore the assessment of the intervention’s effectiveness at changing the HEI, physical activity, sedentary behavior variables, and exploratory measures of BMI and BMI z score by experimental group using Analysis of Co-Variance (ANCOVA).

#### Potential mediators and moderators

Potential mediators include FCCP attitudes, beliefs, perceived barriers and self-efficacy measured on the pre-post surveys. Potential moderators include provider age, ethnicity, race, educational level, income level, years in operation as a FCCP, size of FCCH (number of children in care), accept CACFP or not, and hours of training in the past year.

We will explore the mediational hypothesis, which assumes that interventions change mediators, which in turn change outcome behaviors. We will first assess the experimental group variable (Intervention or Comparison) with the primary outcome behaviors. We will then link the experimental group variable with changes in the proposed mediator (e.g., FCCP’s self-efficacy) and will link proposed mediator with the behavioral outcome. We will repeat these steps looking at all potential mediation relationships hypothesized in the intervention logic model. Clearly, the proposed sample size does not provide sufficient power to test simultaneously all these possible mediators without the risk of capitalizing on chance. However, the information gathered from the individual mediational analyses will provide important preliminary data for generating new hypotheses regarding the intervention’s impacts within FCCH and will guide future intervention research.

For moderation, the GEE models described above will be elaborated by including main effects and interactions with experimental condition of putative moderators of the treatment effect, i.e., provider demographic variables. The additional model terms will allow us to test whether these moderating variables were related to changes in outcomes from baseline to 8 or 12 months, and, further, whether these effects differed by treatment.

#### Dissemination

Upon completion of the Healthy Start Trial, investigators will communicate the results of the trial with the FCCP participating in the trial and more broadly to the FCCP communities of RI and MA through public presentations and discussions. Data will be also published in professional journals. In all dissemination, participating FCCP will not be identified individually; only aggregated data will be presented. Healthy Start data and materials will be made available upon request when reporting and publications have been completed.

## Discussion

Healthy Start is an ongoing cluster randomized trial to study the efficacy of an innovative multicomponent intervention to improve the nutrition and physical activity environments of FCCH and the diet and physical activity of the preschool children cared for in these FCCH. This study builds on the previous work of our research team, which improves the likelihood of success. We have recently documented concerning nutrition and physical activity behaviors of children attending child care in Rhode Island [61, 62], as well as expressed the need for interventions by family child care providers [59, 62]. We have also created the Healthy Start intervention based on lessons learned from previous successful, individually tailored interventions [104–108], especially those focused on children’s food and physical activity [63, 109]. In addition, the design of the intervention and evaluation measures are based on previous work by Co-Investigator, DW [57, 58].

Healthy Start moves the field forward for obesity prevention interventions in child care settings. Few rigorous intervention studies addressing obesity prevention have been conducted in child care settings [110–122]; and most of this previous work has been conducted in centers, not in FCCH. Published intervention studies have not included FCCH, which represent 13% of the spaces available for care in Rhode Island for pre-school aged children and FCCP care for 1.6 million children in the U.S. [123, 124] Only one other randomized controlled trial is currently evaluating an obesity prevention intervention in FCCH [57, 58].

Furthermore, no previously published intervention studies have focused on Spanish speaking child care providers in the US. Our study is reaching a largely Hispanic population of FCCP, including Spanish-speaking FCCP, which have never before been included in intervention studies. Given the obesity disparities observed between Hispanic and non-Hispanic children, reaching providers that care for Hispanic children with obesity prevention interventions is important to reducing such disparities.

Healthy Start also informs the field of obesity interventions in child care settings because of its multicomponent intervention that includes a combination of intervention components not yet been tested in child care settings. The interventions involve: tailored written materials, videos, support coaching using brief MI, and community gatherings. Previous studies in child care have focused on intervention strategies such as food service [112, 114, 120] and curriculum changes [113, 122]; interventions directed toward center-based teachers, such as in-person training [110, 116, 125], non-tailored written materials [116, 119, 122, 125], academic detailing [119], and other types of technical assistance, and strategies that included parent and teacher modeling [121].

Many previous interventions in child care centers have predominantly been delivered by health professionals [57, 58, 65, 93, 94] -- an approach that has neither long-term sustainability nor replicability. Thus, effective, sustainable interventions with FCCPs are needed, and our approach, which uses lay support coaches, has significant promise. Healthy Start utilizes bicultural, community health workers as support coaches (or peer counselors) to assist and empower FCCPs to change their FCCH environments, which is a novel approach for obesity prevention interventions in child care settings. While peer counselors have previously been shown to be effective in changing individual health behaviors in certain populations outside of child care [126–139] and among Hispanic populations [136–142], studies have not adequately evaluated their ability to be effective in child care settings or in fostering environmental change, which is our focus. Additionally, Healthy Start integrates peer counseling with tailored written materials and videos, which is a novel intervention strategy never before studied in a child care setting. We have piloted this approach with families [63, 109]; but only using tailored print materials, not tailored videos, and not in child care settings. These strategies have a high potential to be sustainable, replicable and widely disseminated. The proposed research will expand our knowledge about the efficacy of this innovative approach. Overall, the intervention setting, target population and intervention approaches are all novel. The proposed research will move the frontier of obesity prevention research in child care forward.

Even the most rigorous study designs have limitations that need to be acknowledged. A randomized trial requires FCCPs to volunteer to participate in rigorous study methodologies and the study population will not be a random sample of FCCP, so may lead to selection bias. In particular, having observers come to the home may be a factor in the enthusiasm of providers to participate in the study and may prevent certain providers, i.e., those who may not meet state guidelines, from participating. Another potential limitation is that the intervention is only 8 months long. FCCP select topics to work on each month, with a total of 8 topics addressed over the entire intervention, but they are not exposed to all the topics. Thus, they may not receive all of the information they need to make comprehensive changes to the nutrition and physical activity environment of their FCCH.

However, the strengths of Healthy Start should also be considered. Extensive formative research informed the development of the multicomponent intervention, which is well grounded in behavioral theory and in the culture of FCCH. The intervention utilizes a combination of well-proven traditional, as well as innovative approaches and will be evaluated using a combination of measures including self-report and observational measures of FCCP practices and the environment of the FCCH as well as objective measures of children’s diet, physical activity and weight in a cluster randomized trial, which is the most rigorous research design. Extensive process evaluation will also be conducted, which will help to explain the trial results as well as to inform future intervention studies.

## Conclusions

Thus, the Healthy Start/Comienzos Sanos intervention has a high likelihood of being efficacious and has the potential to be replicated and widely disseminated throughout the US, where improvements in the child care environment are a high priority.

## Additional files

Additional file 1: “Example of a tailored feedback page”. This is an example of the tailored feedback page given to participants during the Healthy Start/Comienzos Sanos study. (DOCX 70 kb)

Additional file 2: “Newsletter Examples: Whole Grains, Healthy Snacks and Fruit (Time)”. This is an example of newsletter pages given to participants during the Healthy Start/Comienzos Sanos study. (DOCX 1785 kb)

## Abbreviations

ANCOVA: Analysis of Co-Variance
BMI: Body mass index
CACFP: Child and Adult Care Food Program
CDC: Centers for Disease Control
CT: Connecticut
DARN: Desire, Ability, Reason, Need
DOCC: Dietary Observation in Child Care
DVD: Digital video disc
EPAO: Environment and Policy Assessment and Observation
FCCH: Family child care home
FCCP: Family child care provider
GEE: Generalized estimating equation
HEI: Healthy Eating Index
MA: Massachusetts
ME: Motivational enhancement
MI: Motivational interviewing
MN: Minnesota
NAP SACC: Nutrition and Physical Activity Self-Assessment for Child Care
NCC: Nutrition Coordinating Center
NDSR: Nutrition Data System for Research
NHANES: National Health and Nutrition Examination Survey
PA: Physical activity
RI: Rhode Island
SAS: Statistical Analysis Software
SCT: Social Cognitive Theory
SNAP: Supplemental Nutrition Assistance Program
UNC: University of North Carolina
US: United States
WIC: Special Supplement for Women, Infants and Children
