# Supplementary material for: Improving nutrition and physical activity environments of family child care homes: the rationale, design and study protocol of the ‘Healthy Start/Comienzos Sanos’ cluster randomized trial
Source: BMC Public Health. 2019 Apr 18;19:419. doi: 10.1186/s12889-019-6704-6 (PMC6472069; doi:10.1186/s12889-019-6704-6)
Supplement: Supplementary file 1 — “Example of a tailored feedback page”. This is an example of the tailored feedback page given to participants during the Healthy Start/Comienzos Sanos study. (DOCX 70 kb) [file 12889_2019_6704_MOESM1_ESM.docx]

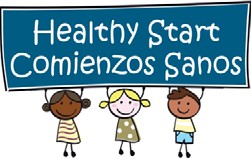
About your child care home…

Jessica, when you answered our survey, you gave us

information about how things run in your home. We also gathered information during the days we spent with you in your home.

With this report, you can see where you are already doing well:
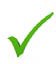
 And where you could make a change: no check mark

**HEALTHY DRINKS**


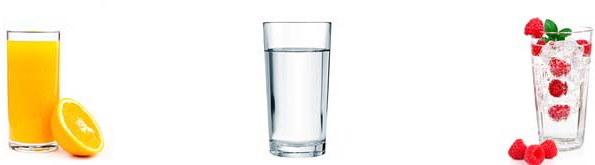


| **BEST PRACTICE:** | **IN YOUR HOME:** | 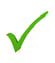 |
| --- | --- | --- |
| **WATER**  Make drinking water available for children at all times. | It appeared drinking water was not available at all times. |  |
| Prompt children to drink water during each indoor and outdoor play time. | It appeared you may not have encouraged children to drink water during active playtimes. |  |
| **JUICE**  Limit 100% fruit juice to no more than two, 4‐6 ounce servings per week (no more than 12oz total per week). | You said you offer 100% juice to children 1 time per month and the serving size you usually offer is 6 ounces. | 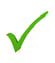 |
| Only serve 100% fruit juice that has no sugar added. | It appeared you did not serve juice that had added sugar. | 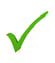 |
