# Supplementary figures and images for: Improving nutrition and physical activity environments of family child care homes: the rationale, design and study protocol of the ‘Healthy Start/Comienzos Sanos’ cluster randomized trial
Source: BMC Public Health. 2019 Apr 18;19:419. doi: 10.1186/s12889-019-6704-6 (PMC6472069; doi:10.1186/s12889-019-6704-6)

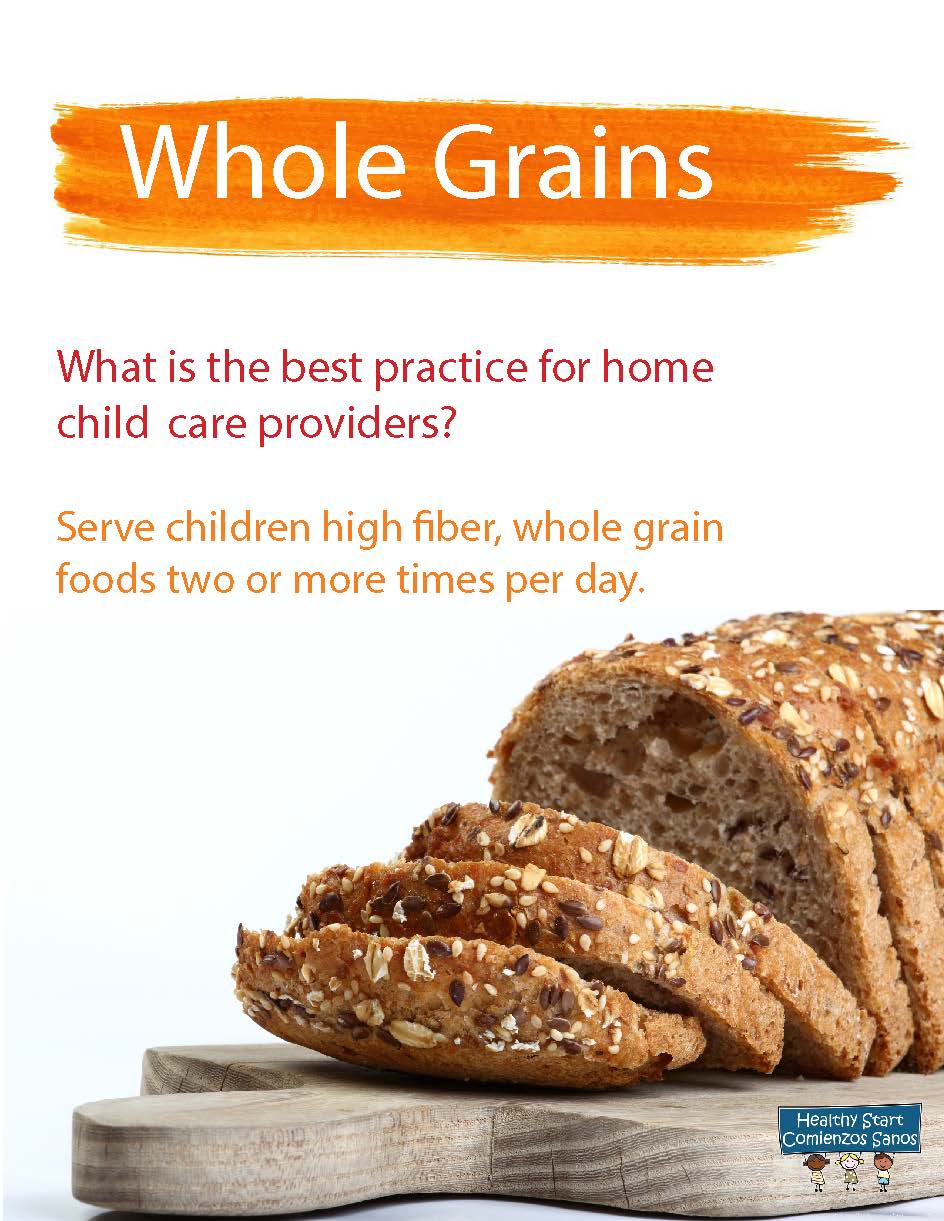


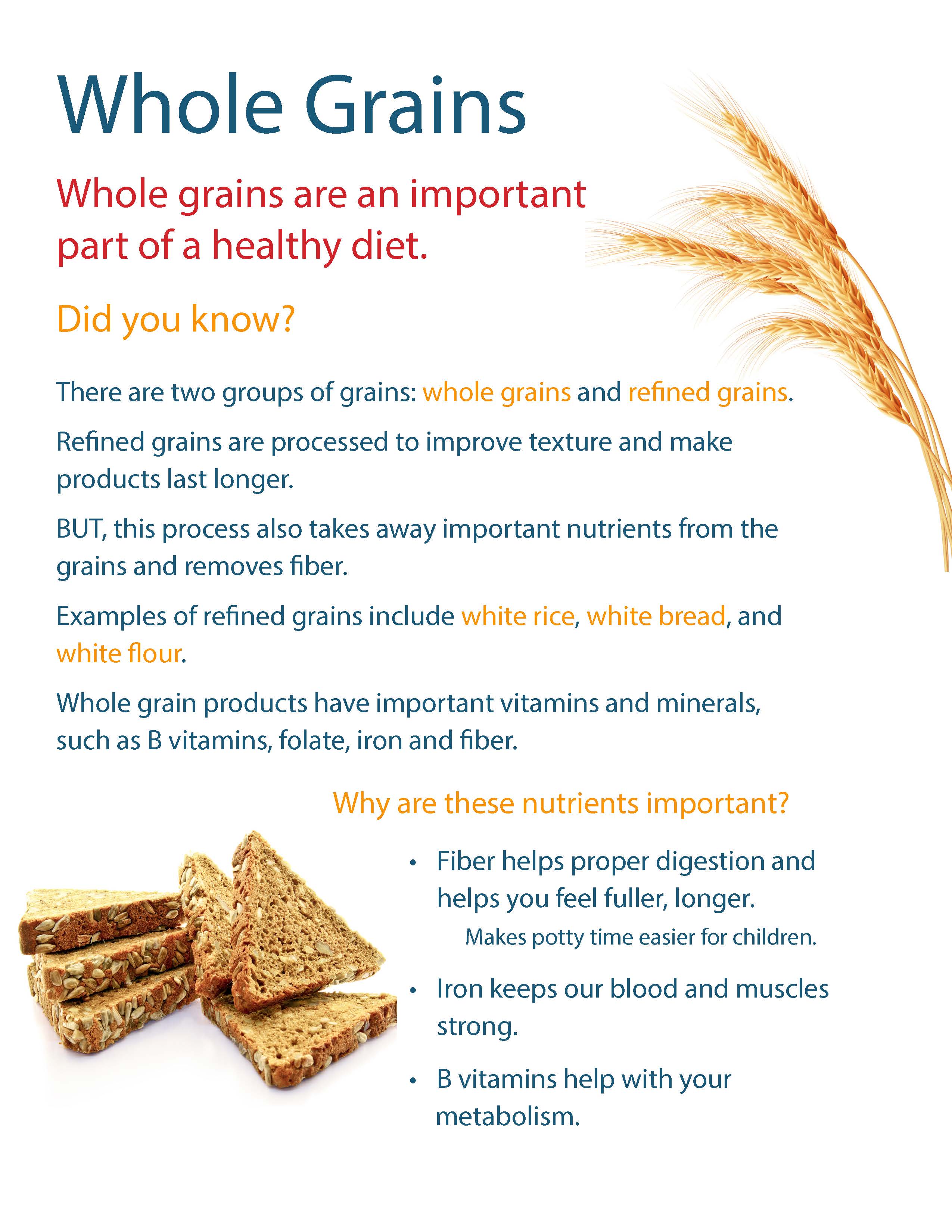


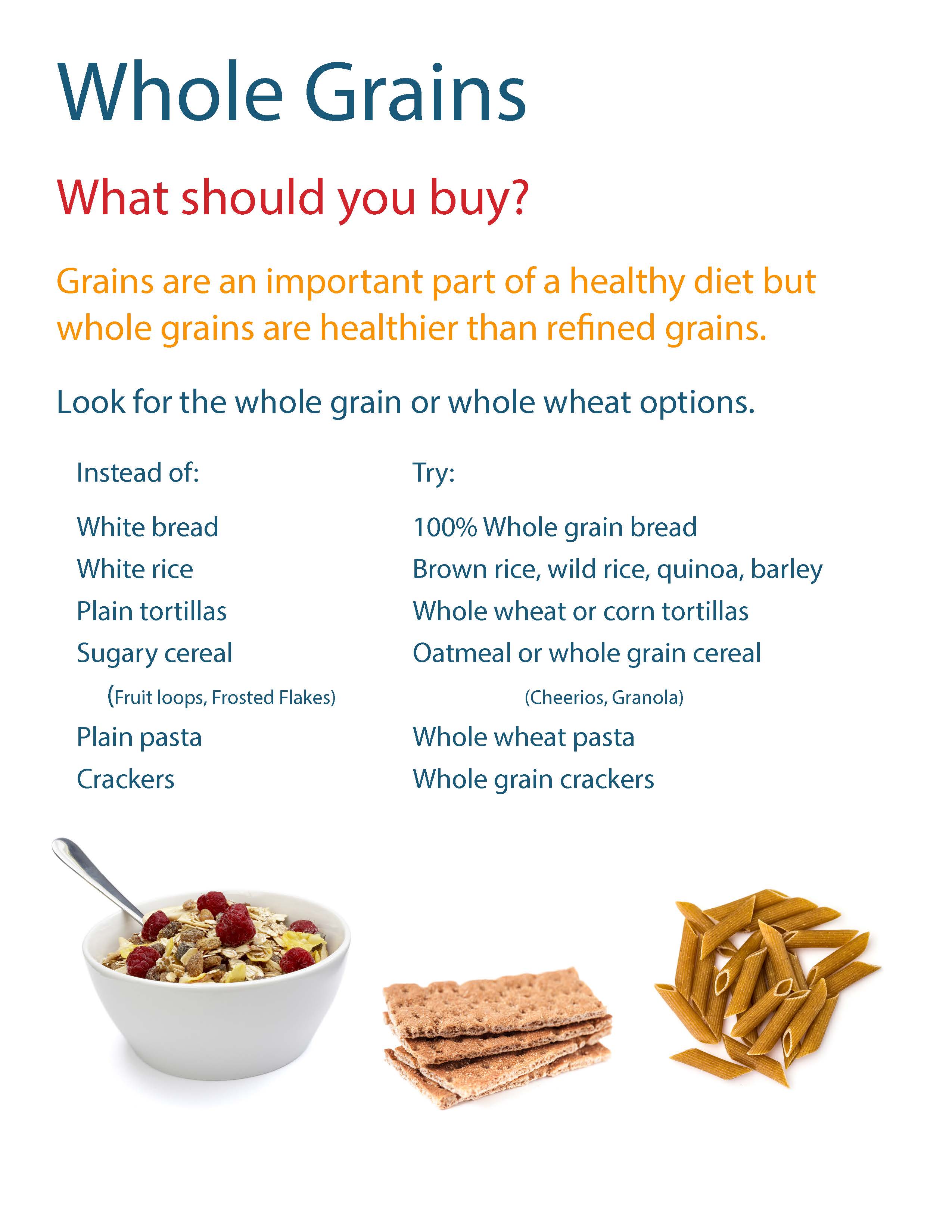


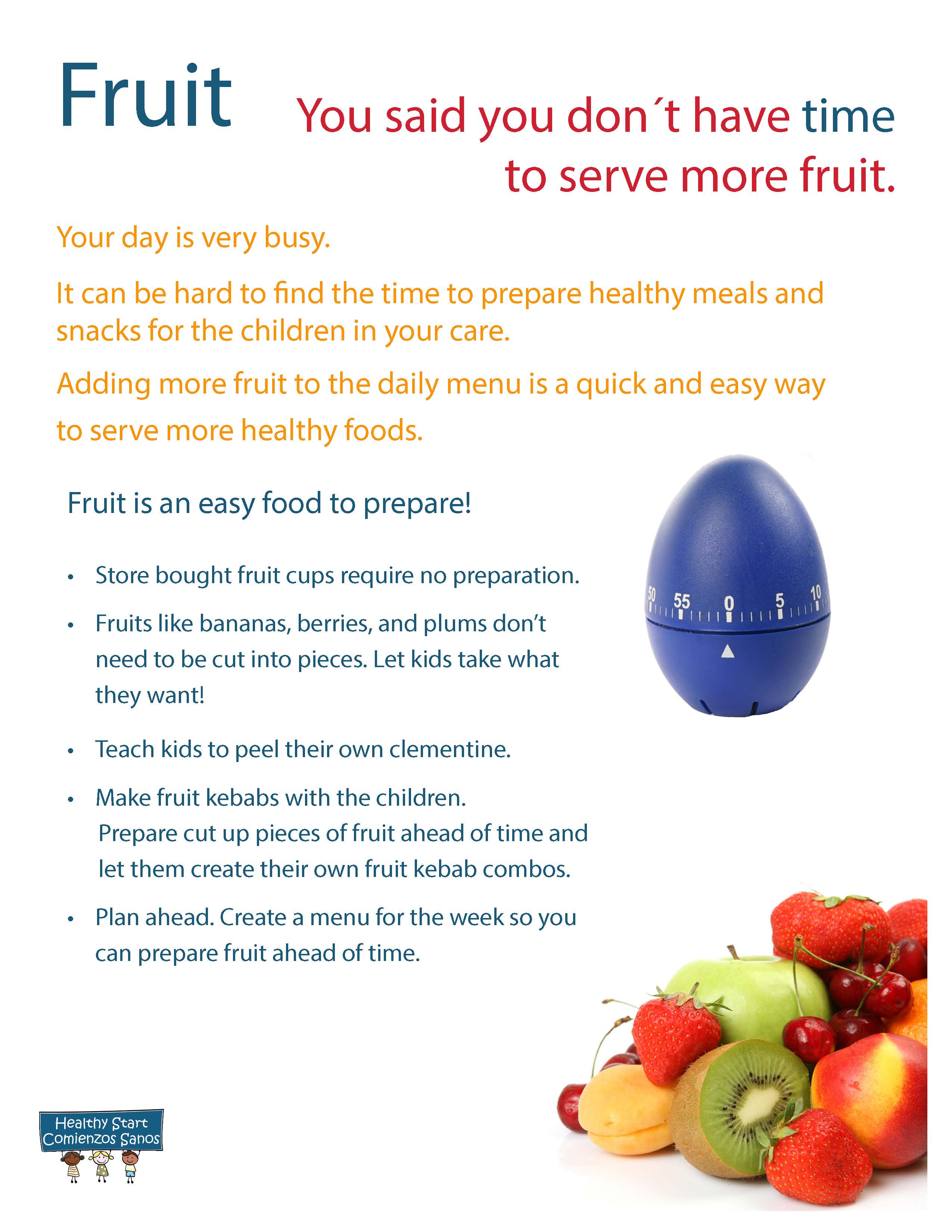


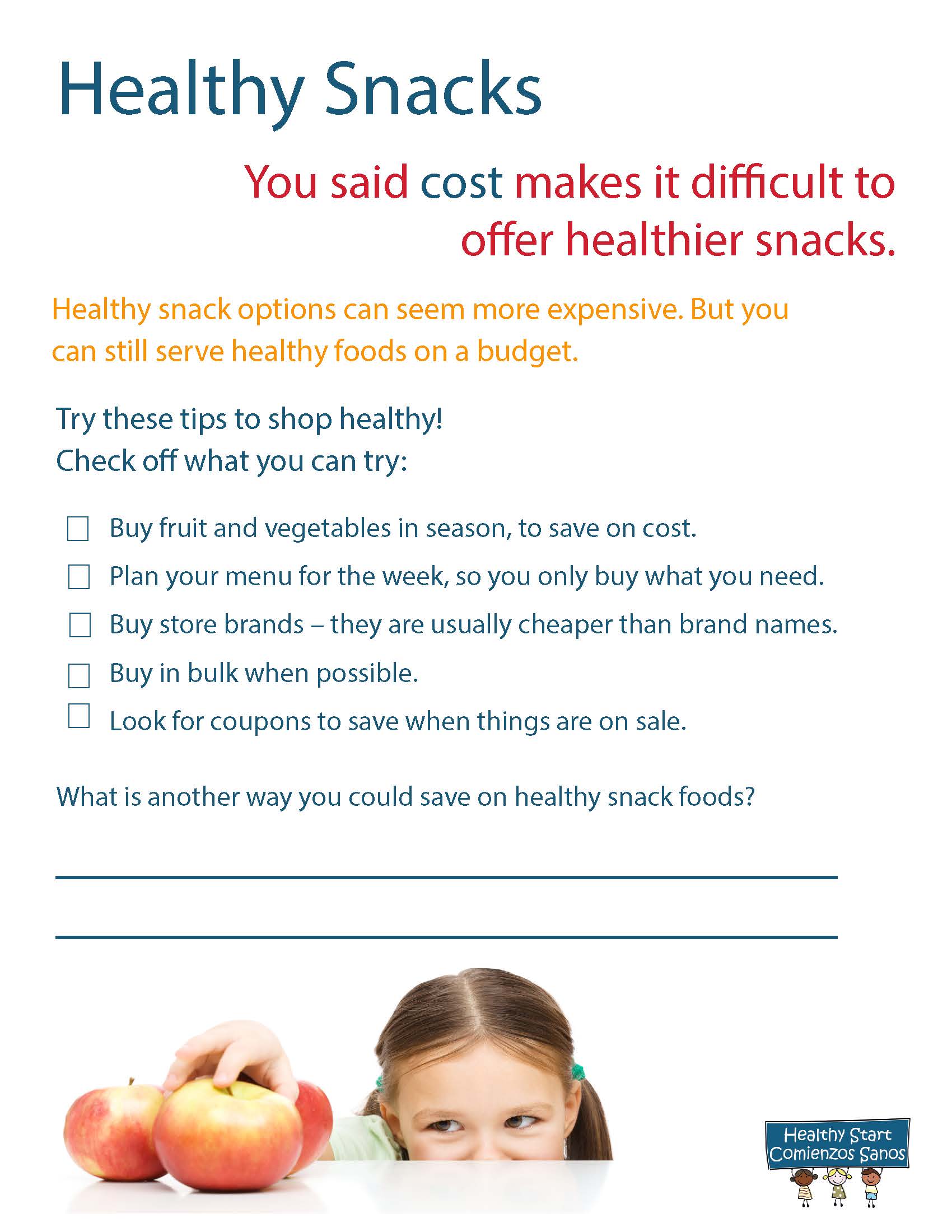

Supplement: Supplementary file 2 — “Newsletter Examples: Whole Grains, Healthy Snacks and Fruit (Time)”. This is an example of newsletter pages given to participants during the Healthy Start/Comienzos Sanos study. (DOCX 1785 kb) [file 12889_2019_6704_MOESM2_ESM.docx]
